# Phage Therapy in Poland – a Centennial Journey to the First Ethically Approved Treatment Facility in Europe

**DOI:** 10.3389/fmicb.2020.01056

**Published:** 2020-06-05

**Authors:** Maciej Żaczek, Beata Weber-Dąbrowska, Ryszard Międzybrodzki, Marzanna Łusiak-Szelachowska, Andrzej Górski

**Affiliations:** ^1^Bacteriophage Laboratory, Hirszfeld Institute of Immunology and Experimental Therapy, Polish Academy of Sciences (HIIET PAS), Wrocław, Poland; ^2^Phage Therapy Unit, Hirszfeld Institute of Immunology and Experimental Therapy, Polish Academy of Sciences (HIIET PAS), Wrocław, Poland; ^3^Department of Clinical Immunology, Transplantation Institute, Medical University of Warsaw, Warsaw, Poland

**Keywords:** phage therapy, phage discovery, phage research, Hirszfeld Institute, Phage Therapy Unit, Polish history

## Abstract

Although phage discovery is an unquestionable merit of the English bacteriologist Frederick W. Twort and the Canadian–French microbiologist Félix d’Hérelle, who both discovered phages over 100 years ago, the Polish history of phage studies also dates back to those years. In contrast to the Western world, developing phage treatment in Poland has never been abandoned despite the country’s tense history marked by the Second World War (WWII) and the communism era. Today, Poland takes a prominent and remarkable place in the phage research area. Furthermore, established in 2005, the Phage Therapy Unit at the Hirszfeld Institute of Immunology and Experimental Therapy in Wrocław, the first such center within European borders, has quickly become a model for other centers in the world facing the issue of widespread antibiotic resistance. This article constitutes an attempt to fill the gap in the scientific literature by providing a comprehensive summary of the long tradition of phage research in Poland.

## Introduction

Recent years have shown a remarkable boost in the development of phage treatment across the globe. The growth of antibiotic resistance sparked a new debate on the problem that has been known for decades and changed the way in which novel therapies are perceived by the public. Such a status of phage therapy did not always seem to be the case. In the first years following phage discovery, phages rapidly lost their initial potential for antibiotics. The success of penicillin postponed the resurgence of phage interest until the next century. The outbreak of the Second World War (WWII) increased the value of penicillin. At that time, the major goal was to have an adequate supply of the drug. Production of penicillin in the United States alone jumped from 21 billion units in 1943 to more than 6.8 trillion units in 1945. As of March 1945, penicillin was widely available at pharmacies across the States (Aldridge and Sturchio, 1999). Hence, one may be surprised that phages had been used in medicine 10 years prior to the discovery of penicillin. However, not all countries rejected phages in favor of penicillin. In such a way, Eastern Europe became the only part of the world where phage therapy was considered a serious alternative to antibiotics. Even just a few years ago, the “phage therapy” term was still barely noticeable in PubMed search results, indicating that this resurgence is still an ongoing matter (Pirnay et al., 2019).

Early research on phage, which was by definition cheaper and limited to basic microbiological assays, constituted a great opportunity for the less wealthy and less developed part of Europe. Surely, that opportunity has been fully realized. Among the Communist states, Poland and Soviet republics (particularly the Georgian and Russian SSR) are best known for their significant contribution to the development of phage treatment. On the other hand, in the West, inadequate and poor methodology contributed to the perception of phages as a failed concept. Such discrepancies between the Western and Eastern parts of Europe are probably the best explanation as to why countries on the east side of the iron curtain have become pioneers in research on phages and their therapeutic connotations.

Since 2005, a year after Polish accession to the European Union, phage therapy in Poland has changed dramatically becoming a model way of treatment for other centers in the world (Gill and Young, 2011). The establishment of the Phage Therapy Unit (PTU) at the Hirszfeld Institute of Immunology and Experimental Therapy in Wrocław, Poland, coincided with a sharp increase in morbidity and deaths in the world caused by antibiotic-resistant superbugs. The beginning of the 21st century left little illusions regarding the future of antibiotics. The danger of multidrug-resistant (MDR) strains has even been emphatically entitled the “Battle against antibiotic resistance is being lost” in the pages of *Lancet* (Morris, 2007).

Over the years, the phage center at the Hirszfeld Institute has been highlighted by numerous prestigious publications, such as Science or Nature Medicine (Stone, 2002; Hausler, 2006). Hausler described vivid examples of patients who could officially seek a phage treatment at Wrocław clinic years before it became a popular subject in the Western mainstream media. In 2004, the leading national German daily newspaper *Die Welt* published an article about Polish phage therapy conducted at the Hirszfeld Institute entitled “*Viren als Verbündete gegen Infektionen*” [Viruses as allies against infections] with the conclusion that “Poland has taken on the international leadership in clinical phage therapy in the last decades” (Bettge, 2004). These efforts cannot be underestimated in the era of rapidly growing phage biotech startups that call themselves “clinical-stage” companies despite their little scientific background, experience in phage treatment in humans, and no significant achievements. Keen, in his article summarizing a century of phage research, predicts that in the future phage-based therapies will become widely used in medicine and agriculture (Keen, 2015).

However, in the following sections, we will focus solely on the past and try to present the rich and fruitful centennial history of phage therapy on Polish soil, a story that dates back to regaining independence by Poland after 123 years of its partition and loss of independence. We believe that Polish accomplishments in this field deserve proper attention in the history of phage research and therapy.

## The Interwar Period

The first article on phage therapy was published in French in 1921 and was focused on staphylococcal skin infections (Bruynoghe and Maisin, 1921). At that time, Poland was a young independent state trying to re-establish its statehood in a new reality after the First World War (WWI) and after 123 years of being wiped off the map of Europe. The Second Polish Republic was the sixth largest country in Europe inhabited by nearly 30 million people. In contrast to the Soviet Union, shortly after WWI, Poland’s significance in the world was rather limited. Thus, one can safely say that the beginnings of the Eliava Institute in Tbilisi, Georgia, which was founded in 1923, were quite different from the situation of Polish scientists. Institute Pasteur served as a model for building Soviet bacteriology. Furthermore, an extraordinary role in the development of the Eliava Institute was played by famous scientist Felix d’Hérelle. The founder of Georgian Institute, Prof. George Eliava worked with d’Hérelle in Paris in the years 1918–1920. Thanks to that relationship, Eliava had direct access to ground-breaking phage work (Myelnikov, 2018). In short, Polish microbiologists could not compete with a well-prospering Eliava Institute. The Institute, during its best times, employed approximately 1,200 researchers and support personnel and produced phage preparations (often several tons a day) against a dozen bacterial pathogens (Sulakvelidze et al., 2001). The tables were to turn almost a century later when Poland joined the European Union in 2004. Despite such inequalities in the situation of both countries, it would be a mistake assuming that Polish achievements in the phage field were meaningless. The first Polish work on phage treatment in controlling dysentery was published by the medical periodical *Lekarz Wojskowy* [Military Physician] in 1923 (Kalinowski and Czyż, 1923). It was just a few years after d’Hérelle’s pioneer attempt to treat dysentery with phage in 1919 (Dublanchet and Fruciano, 2008).

The Polish microbiologist Prof. Władysław Kunicki-Goldfinger sheds more light on Polish phage scientists who actively published their articles in the interwar period (Kunicki-Goldfinger, 1992). He recognizes such names as Fejgin, Lipska, Łomiński, Sierakowski, and Szymanowski, among others. Particularly, Dr. Irena Lipska deserves further attention as her work has been cited by others several years after her publishing activity (Kennedy et al., 1986; Hsu et al., 2002). In 1931, she presented her findings at the 9th International Dairy Congress in Copenhagen, Denmark. Her abstract entitled “d’Hérelle phenomenon in milk” focuses on phage presence in milk and their possible antibacterial action in it, a phenomenon that is still of high importance among scientists (Lipska, 1931). She continued her research in the following years (Lipska, 1937). Dr. Lipska was also responsible for supplying the Ujazdowski Hospital in Warsaw with phage preparations that were later used during WWII in the years 1940–1944 (Letkiewicz et al., 2017).

### Bronisława Fejgin

The outbreak of WWII brought a real tragedy into the lives of all Poles and the scientific community was no exception. Balińska and Schneider in a translation of Prof. Ludwik Hirszfeld’s autobiography entitled “Ludwik Hirszfeld: The Story of One Life” quote Hirszfeld’s memories from that time (Balińska and Schneider, 2010). Prof. Hirszfeld lists the names of several Polish bacteriologists who died, were tortured, or were killed during WWII. Among many others, the name of Bronisława Fejgin is listed, who was a member of the team responsible for developing an outstanding method of testing water for the presence of paratyphoid germs. It is not a well-known fact that one of the first Polish references to phages comes from her. Bronisława Fejgin was a Polish-born Jewish physician. In 1914, she graduated from Sorbonne Medical School in Paris. Thus, most of her work was published in French (Fejgin, 1923, 1936; Fejgin et al., 1927). Her legacy in the field of bacteriology and serology is of ongoing relevance (Weisz and Grzybowski, 2016). In 1926, Bronisława Fejgin developed the laboratory typhus identification method which was later clinically implemented by Ludwik Fleck in 1942 in the Lwów Ghetto (Weisz and Grzybowski, 2011, 2016). The importance of her achievements in the phage field was already recognized in 1928 by Klosterman and Small (1928). The authors mentioned her name in the first sentence of their publication along with such prominent names like d’Hérelle and Blair to emphasize her success in isolating a lytic phage against bacteria classified then as *Bacillus diphtheriae* (today *Corynebacterium diphtheriae*). What might be even more unheard for today’s phage researchers, Weisz and Grzybowski suggest that the discovery of phage by Twort and d’Hérelle was, at least partially, based on Fejgin’s preliminary research as she described a lytic agent that would later become known as a phage. At this point, it must be added that she never claimed such achievement for herself and fully respected d’Hérelle’s pioneer discovery. In 1927, in her article published entirely in Polish she describes “d’Hérelle’s phenomenon” as the main driving force responsible for bacterial lysis (Fejgin, 1927). In this article, she predicted with great accuracy the future of phage research, concluding that “facts related to the discovery of invisible bacterial forms (…) are ready to shake faith in the immutability of bacterial species.” Her legacy is also unique for another reason. Back then, the scientific world was mostly dominated by male researchers. These two facts considered together lead two the obvious conclusion that Bronisława Fejgin deserves a significant recognition from not only phage researchers but also the entire scientific community. There is no doubt her achievements would have been even more significant if not for her tragic death in the Warsaw Ghetto in January of 1943. Currently, it is very hard to find her complete scientific work in the form of publications but Fejgin appeared repeatedly in articles after WWII. For instance, Coetzee and Sacks emphasize in the prestigious journal Nature her role in detecting a lysogenic strain of *Proteus* (Coetzee and Sacks, 1959).

### Jerzy Jasieński

Around the same period, Dr. Jerzy Jasieński from Jagiellonian University in Kraków used phage lysates to combat staphylococcal purulent infections in a group of 40 patients. He revealed his results in 1927 in the pages of *Polska Gazeta Lekarska* [Polish Medical Journal] entitled “*Próby zastosowania bakteriofagji w chirurgji*” [Attempts to use bacteriophagy in surgery] (Jasieński, 1927b). In his research, 75 out of 85 collected *Staphylococcus* strains were sensitive to phages that had been isolated by him from patients’ infection sites. However, Jasieński was skeptical in terms of subcutaneously injected *Staphylococcus aureus* phage due to the unsatisfactory clinical outcome of applied treatment (among 11 patients with bone infections and chronic furunculosis only two cases indicated full recovery). Although his methodology does not meet today’s standards, he recognized back then that phage preparations are generally safe for patients with no evident correlation between clinical outcome of phage treatment and the response of patients’ immune system as reflected by anti-phage antibodies. The work described above is not the only article Jasieński published in 1927. His review paper from the same year entitled “*O Bakteriofagji*” [About Bacteriophagy] constitutes a remarkably comprehensive and detailed summary of the then state of knowledge about phage therapy in the interwar period (Jasieński, 1927a). On 30 pages, the results of phage authorities such as d’Hérelle, Gratia, Arkwright, Preisz, and Eliava were in detail evaluated and described by Jasieński. In the summary, he concludes that *in vitro* results obtained in the laboratory setting should not be directly translated into the human model. It is hard not to notice that such rules of evidence-based medicine (EBM) are important especially nowadays when articles determining the favorable outcome of phage treatment based only on *in vitro* tests or animal models are being published extensively almost on a daily basis.

## The Postwar Period

The atrocities of WWII do not need closer attention here as this subject has been visited countless times and is still vivid in the memory of many people. Prof. Ludwik Hirszfeld in his autobiography writes about “immensely difficult circumstances” under which Polish scientists were forced to work in postwar Poland (Balińska and Schneider, 2010).

Despite such historic turbulences, phage treatment was still conducted by Polish physicians. An interesting example can be found in *Dzienniki Powojenne* [Post-war Diaries] by the famous Polish writer Maria Dąbrowska, published posthumously in 1996 (Dąbrowska, 1996). This personal collection of writer’s memories provides a unique insight into a rough Polish history in the 20th century. The author mentions about her struggles with a purulent pelvic inflammatory disease that lasted for 4 months in 1942. It was then that she was treated by phages against *Escherichia coli*. Citing her own words, “Dr. Czubalski cured me (of the disease) starting from a four-time blood transfusion and then by applying phage provided by Dr. Kryńska along with his own methods of eliminating inflammation caused by *E. coli.*” She adds that Dr. Czubalski literally saved her life when other doctors from the university clinic had given up on her. Unfortunately, Maria Dąbrowska does not provide any more details about the therapeutic use of phages, but even this short section constitutes an important contribution to the whole picture of Polish medicine with phage as a recognized method of fighting bacterial infections in the most severe cases. It is worth mentioning that Maria Dąbrowska was already quite famous back then and the use of phages could be an expression of the highest concern for the patient.

During the period following the end of WWII, research on the therapeutic application of phages continued. In 1951, Szczepańska on the pages of *Pediatria Polska* [Polish Journal of Pediatrics] published her preliminary results on the preventive application of phages in the newborns (Szczepańska, 1951). In the same journal, Lipska published her article in which she described her experience with phage therapy in infants during the war and early postwar period (Lipska, 1951). We learn from this article that at least 100 Polish physicians applied phages during the German occupation, when other antibacterials (i.e., sulfonamides) were not available. Certainly, she was responsible for the introduction of this treatment method in pediatric health care centers in postwar Warsaw. In the subsequent years, Dr. Irena Lipska was involved in the use of phage therapy in patients with typhoid infections.

Interestingly, recently published review on the history and development of phage therapy in Brazil describes work of aforementioned Dr. Irena Lipska (De Freitas Almeida and Sundberg, 2020). The authors cite her article published during the postwar period. In 1950, Lipska published a recommendation to the Brazilian medical community on the prophylactic use of phages to protect newborns against diarrhea (Lipska, 1950). Given as the first liquid after birth, phages were found to be “simple and harmless.” The article was published in Portuguese. In June 1950, Lipska participated in the international conference in Rio de Janeiro, so the publication in Portuguese is probably related to this event.

### Ludwik Hirszfeld

When analyzing phage research on Polish soil in the postwar period, it is impossible not to mention Prof. Ludwik Hirszfeld. His indisputable achievements include naming of the blood groups (A,B,AB, and 0) along with the discovery of their heritability and the discovery of serological conflict between mother and child, which was later confirmed by the presence of the Rhesus (Rh) factor. Hirszfeld was an honorary doctor of the universities of Prague and Zurich and in 1950 was a candidate for the Nobel Prize, which he ultimately did not receive, probably for solely political reasons (Kucharz et al., 2010). The’50s of the XX century was a period of the highest intensification of the so-called Cold War, which may have affected the decision of the Nobel Committee (Kozuschek, 2005). Obviously, these are not all of Hirszfeld’s achievements. During WWI, he conducted vaccination programs and fought the epidemics of spotted fever, typhoid fever, and malaria in the south of Europe. Over this period, Hirszfeld isolated a strain of typhus bacillus that was later named to honor him as *Salmonella hirszfeldi*. He found out that the strain was an important etiological agent of typhoid, and he considered that it might have been the cause of the epidemics (Lonc and Gościniak, 2012).

However, for the phage community, the most crucial research seems to be Hirszfeld’s research on phages he conducted right after WWII. Contrary to Bronisława Fejgin, Hirszfeld together with his wife Hanna and daughter Maria survived the Warsaw Ghetto, which they left in 1942. After the tragic death of their daughter in 1943, both spouses lived in secret in the village Wesoła near Warsaw until the end of the war (Jasińska, 2014).

**FIGURE 1:**
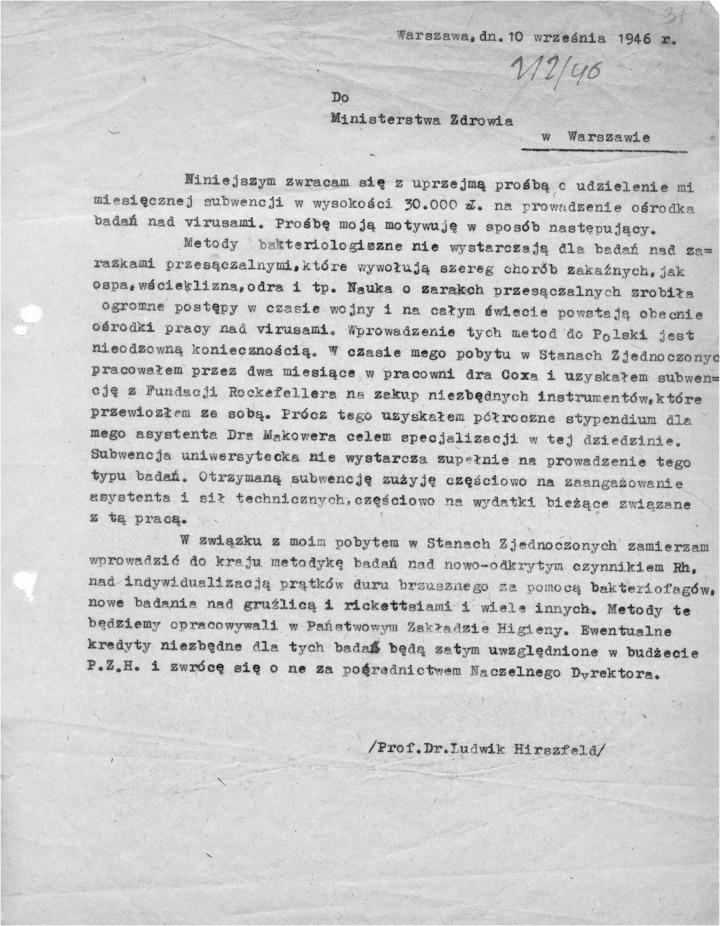
Prof. Ludwik Hirszfeld’s letter to the Ministry of Health dated September 10, 1946 [in Polish]; source: Hirszfeld Institute’s archive.

On August 1, 1945, Hirszfeld went to Wrocław, a city located within the newly created Western borders of Poland. He was involved in organizing the first Faculty of Medicine, which was established in the same year as one of the six faculties of the University of Wrocław and Technical University. He also established the Department of Medical Microbiology, which was later transformed into the Institute of Immunology and Experimental Therapy of the Polish Academy of Sciences. An invaluable source of Hirszfeld’s activity in the first years after arriving in Wrocław constitutes his extensive correspondence kept in the archives of the Hirszfeld Institute. The collection includes numerous letters written by and to Hirszfeld. From that correspondence, we learn that in September 1946, roughly 1 year after the end of WWII, Hirszfeld applied to the Ministry of Health for funding amounting to 30,000 Polish Zlotys. He motivated his request with the necessity of conducting research on phage against typhoid fever pathogens among others that could be conducted at the PZH [*Państwowy Zakład Higieny* (State Department of Hygiene)] (Figure 1).

In the same year, Hirszfeld visited Prof. James Craigie in Toronto. Hirszfeld was extremely impressed by his work on phage. He noted phage narrow specificity against bacterial typhoid pathogens and praised the significance of such a phenomenon for medicine (Balińska and Schneider, 2010). He returned from this trip with selected phages and their bacterial hosts with the intention of passing them to individual branches of the PZH. In a letter to the Director of PZH, Prof. Feliks Przesmycki, dated June 1947, Hirszfeld informed him that the phages he brought to Poland had been propagated and he planned to organize an additional phage typing course later that year. Hirszfeld continued his work on phage in the Wrocław branch of the PZH, which he described in August 1947 in a letter to Dr. Zygmunt Grynberg, the Director of the Organizational Department of the Ministry of Health. We learn from that letter that methodology regarding phage typing against typhoid fever bacteria had been developed by the Hirszfeld team. Furthermore, Hirszfeld announced that the International Committee in Copenhagen had chosen the Wrocław branch of the PZH as the national phage research center in July 1947.

**FIGURE 2:**
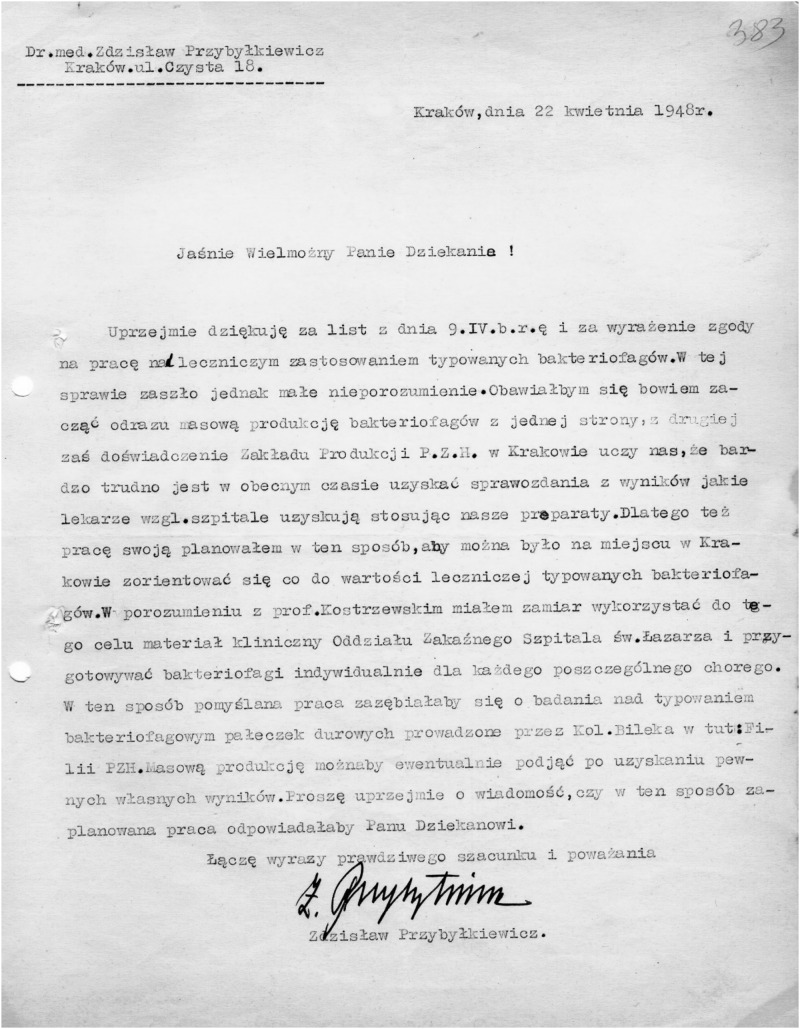
A letter of the director of the Medical Microbiology Institute at the Jagiellonian University in Kraków, Dr. Zdzisław Przybyłkiewicz to Prof. Ludwik Hirszfeld dated April 22, 1948 [in Polish]; source: Hirszfeld Institute’s archive.

Ludwik Hirszfeld played a leading role in the initiation of phage therapy in Poland after WWII. In a letter to Hirszfeld dated 22 April 1948, Dr. Zdzisław Przybyłkiewicz, then the director of the Medical Microbiology Institute at the Jagiellonian University in Kraków, expresses his gratitude for allowing him to use phages for therapy and points to the difficulties in obtaining feedback on the results of phage treatment carried out in different hospitals. In view of those difficulties, Przybyłkiewicz suggested that the therapy could be continued at the infectious ward of the St. Lazarus Hospital in Kraków using personalized phages prepared by his team (Figure 2). This approach could strengthen efforts to obtain reliable data on the safety and relative efficacy of phage therapy. Regretfully, we were unable to identify any publications related to this effort and the untimely death of Hirszfeld caused a temporary regress in further developments of phage therapy. At any rate, this incident confirms that Hirszfeld tried to expand phage therapy to other Polish cities, while the role of Dr. Zdzisław Przybyłkiewicz in the promotion of that therapy also needs to be recognized. Notably, Przybyłkiewicz established the Vaccine Production Plant in Kraków that initiated production of phage preparations and was later transformed into IBSS BIOMED S.A., the company which continues interest in phage production to this day (the company’s history is available at https://www.biomed.pl/en/company/history/).

In the following correspondence with the Deputy Minister of Health, Jerzy Sztachelski, Hirszfeld stated that the methodology of phage typing of typhoid fever bacteria had spread to six branches of the PZH throughout Poland with the intention to include all branches by the end of 1949 (Meissner, 2012). What clearly emerges from these letters is a picture of a man who was truly devoted to his research and was able to effectively fight for funds during the rough postwar period.

On March 7, 1948, Hirszfeld gave a lecture at the meeting of the Wrocław Scientific Society entitled “*Walka świata niewidzialnego z pozawidzialnym*” [The battle of the invisible with the imperceptible] in which he fully emphasized the antibacterial potential of phage by saying “Phage act in such an unbelievable small quantity, they destroy bacteria so completely, their strength exceeds the body’s immune capabilities” (Hirszfeld, 1948). One month later, in April 1948, he published an article in *Polski Tygodnik Lekarski* [Polish Medical Weekly] entitled “*Bakteriofagi i ich rola w rozpoznawaniu duru brzusznego*” [Phages and their role in the diagnosis of typhoid fever], where he continued his flattering opinion about phage and their specificity of action (Hirszfeld et al., 1948). In his book *Immunologia ogólna* [General Immunology] from 1949, he added, “The antibacterial activity of the immune system is much weaker than bacteriophages. The practical implications of this phenomenon should be further explored” (Hirszfeld, 1949). In a letter from 1950 to Prof. Feliks Przesmycki, Hirszfeld emphasized that phage research should be conducted at all Departments of Medical Microbiology in Poland (Meissner, 2012). We truly believe that Hirszfeld’s words and actions deserve special attention as they took place at a time when phage research was almost completely abandoned in the countries west of Poland and no one paid serious attention to the potential of phage treatment. Hirszfeld was fully aware that his research must create a scientific basis for improving human health and that was the mission he was called to pursue (Bajer, 2003).

### The Beginnings of the Hirszfeld Institute

As mentioned above, in December 1952 pursuant to Resolution No. 70 of the Polish Academy of Sciences, the Department of Medical Microbiology in Wrocław was transformed into the Institute of Immunology and Experimental Therapy. Naturally, Prof. Ludwik Hirszfeld became the first Director as his efforts led to the establishment of the Institute. He was not only the founder and first director. Hirszfeld shaped the scientific profile and activity of the Institute for the coming years. The Resolution of the Presidium of the Polish Government dated 1954 on the establishment of the Institute included plans for conducting research on lytic activity of phage. That Resolution was prepared mainly on the basis of Hirszfeld’s previous work (Meissner, 2012). Officially, the Institute started its activity in February 1954, 1 month before Hirszfeld’s death. The Institute was named after his founder, and these days it is internationally recognized under the name of the Hirszfeld Institute. Ludwik Hirszfeld was aware of the Institute’s position and importance by describing it as the only one of its kind in Poland and significant among the world’s medical and scientific institutions (Bajer, 2003). Several years later, Sulakvelidze et al. (2001) concluded that the most detailed studies published in English on the use of phages in clinical settings have come from the Hirszfeld Institute.

In the beginning, the Institute was situated in the old building of the Department of Medical Microbiology. An extremely important event in the history of the Institute was the construction of the new facilities located on 8 ha in the south of Wrocław. Its completion at the beginning of 1975 allowed all laboratories to be gathered in one place, which until then had been scattered all over the city. It also allowed an increase in and expansion of the scientific activity of the Institute. The new Institute’s headquarters was a result of persistent and untiring efforts by Prof. Stefan Ślopek, the second director of the Institute (Duś and Ługowski, 1996).

### Stefan Ślopek

After the death of Ludwik Hirszfeld, in September 1954, the aforementioned Prof. Ślopek, former Head of the Department of Clinical Microbiology of the Silesian School of Medicine, became the Director of the Institute (Duś and Ługowski, 1996). He served this role for 31 years remaining the longest-serving Director of the Hirszfeld Institute. During his directorship, Prof. Ślopek contributed greatly to the expansion of phage therapy in the form that was possible in his time. The Institute expanded his phage bank and produced phage preparations (phage lysates) directed against various pathogens. Those preparations were then distributed mainly among local hospitals and outpatient clinics that were supposed to give feedback on the results of treatment. The treatment in such a form involved more than 1000 patients. Prof. Ślopek’s team published many articles, mainly in the local journals, reporting very high success rates (oscillating around 90%) for the results of phage therapy Ślopek et al., 1983a, b, 1984, 1985a, 1985b, 1985c,1987; Kucharewicz-Krukowska and Ślopek, 1987; Weber-Dąbrowska et al., 1987). Moreover, those reports have emphasized the safety of the therapy and very few side effects. Although today’s analysis of phage therapy does not match those success rates, it should be kept in mind that most of the patients treated in the past were acute cases of bacterial infections that are more susceptible to therapy than chronic patients whose infections are much more difficult to control (Międzybrodzki et al., 2012). In addition, Ślopek’s team did not personally monitor the patients but had to rely on other centers’ reports with all the possible shortcomings of this type of “remote” evaluation.

**FIGURE 3:**
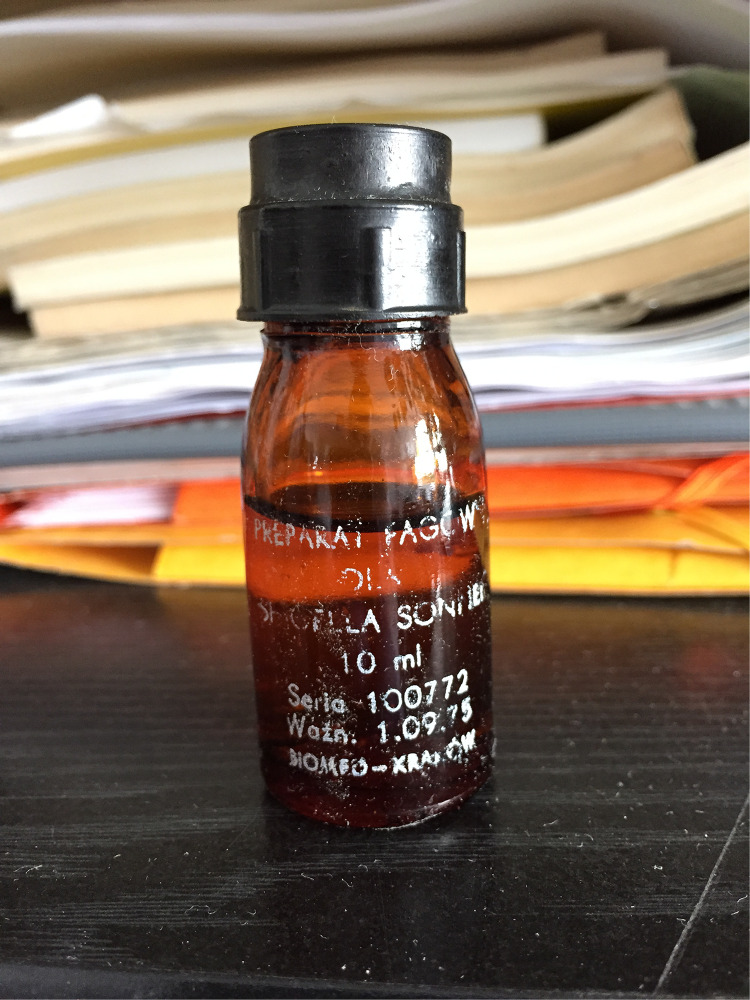
A 10-mL ampoule with *Shigella sonnei* phage cocktail intended for oral administration. Phage cocktail was manufactured by BIOMED in Kraków (July 10, 1972) according to the instructions developed at the Hirszfeld Institute. Source: Private archive of Maciej Żaczek.

Special attention should be given to the research on phage against *Shigella* species causing dysentery (*Shigella sonnei* and *Shigella flexneri*), which was extensively developed at Hirszfeld Institute at that time (Mulczyk and Kucharewicz-Krukowska, 1957; Metzger and Mulczyk, 1958; Ślopek et al., 1961, 1968a,b; Mulczyk et al., 1967; Krzywy, 1971; Mulczyk and Ślopek, 1971; Kostrzewski et al., 1974). Dysentery was a significant epidemiological problem in the 1960s and early’70s in Poland and other eastern countries, especially among children. In 1971, the Reference Dysentery Center was established at the Hirszfeld Institute. The Center was responsible for collection from various European health centers of over 5,000 *Shigella* strains that were later tested for their phage sensitivity. This work resulted in the formation of an international set of phages for typing *Shigella* species and served as a basis to create polyvalent phage cocktails for prophylaxis of *S. sonnei* and *S. flexneri* infections. Such a phage cocktail was widely applied for epidemiological purposes to suppress dysentery outbreaks. Mulczyk and Ślopek reported the production of more than 1,000 L of polyvalent specific phage preparations by the Kraków Serum and Vaccine Laboratory for use in children’s institutions in 1972 (Mulczyk and Ślopek, 1974). Notably, research on *Shigella* phage spread throughout other centers in Poland such as Gdańsk, Katowice, Łódź, Poznań, and Warsaw (Gawronowa et al., 1974; Kokocińska and Rokossowski, 1974; Nowak-Lipińska and Libich, 1974; Szkudlarek, 1974). Among phages deposited in collection of the Hirszfeld Institute, there are still few historic ampoules with *Shigella* phage cocktails from that period (Figure 3).

Further, Kańtoch and Mordarski published their preliminary and pioneer results on phage affinity to mouse cancer cells *in vitro*. The authors hypothesized that some of the cancer cells could contain active phage virions inside them (Kańtoch and Mordarski, 1958). One of the topics investigated at Hirszfeld Institute was an immunogenic effect of phage preparations on the induction of anti-phage antibodies in patients undergoing treatment (Kucharewicz-Krukowska and Ślopek, 1987). This area of research has been greatly expanded at the Hirszfeld Institute. In addition, Kańtoch investigated various aspects of phage phagocytosis (Kańtoch et al., 1958).

Noteworthy is also the series of articles published in the 1960s by Anna Przondo-Hessek from the Medical Academy in Wrocław (today: Wrocław Medical University) which covers the morphology, biology, and therapeutic applications of *Klebsiella* phage from her collection (Przondo-Hessek and Ślopek, 1967a, b; Przondo-Hessek et al., 1967).

### Warsaw Phage Treatment

In the 1950s and 1960s, Wrocław was not the only phage center in Poland. Besides aforementioned research on phage against *Shigella* species, therapeutic connotations of phage were investigated by Dr. Michał Lityński whose detailed work dedicated to *E. coli* phages in the treatment of bacterial infections such as dysentery and renal pelvis inflammation was published in 1950 by Polish journal *Medical Review* [Przegląd Lekarski] (Lityński, 1950). Lityński focused on his experience gained during WWII and the findings of other authors. Similar to the work of Dr. Jerzy Jasieński, described above, Dr. Lityński’s critical approach is noticeable. He emphasizes the necessity of close cooperation between bacteriologist and physician in order to achieve valuable data and increase the chances of a favorable outcome of treatment.

In the Medical Academy of Warsaw (today: Medical University of Warsaw) Witoszka and Strumiłło applied phages to surgical wounds infected by antibiotic-resistant *Staphylococcus* strains (Witoszka and Strumiłło, 1961). The authors were fully aware of increasing antibiotic-resistance, noting that nearly 100% of coagulase-positive staphylococcal strains were already resistant to penicillin and streptomycin. Phage preparations were provided by the Sanitary and Epidemiological Station in Warsaw and its Phage Unit run by a longtime phage specialist Irena Lipska about whom we wrote earlier. In the years 1960–1962, among 50 patients who underwent phage treatment, a good clinical outcome (including pathogen eradication) was obtained in 38 of them. Interestingly, phage preparations were applied in the form of an aerosol.

## Democratic Transition in Poland

In 1989, Poland once again in the XX century had to face political and economic challenges. It was the first country from the so-called Eastern bloc to start a democratic revolution that eventually led to the fall of the Berlin Wall and the creation of a whole new Europe. Despite numerous obstacles, both financial and political, phage treatment at Hirszfeld Institute was not interrupted. The following years brought new articles focused on the promising clinical outcome of phage therapy in humans published both in Polish and in English (Weber-Dąbrowska et al., 1996, 1997, 2003). From the Institute’s brochure published in 1996 we learn that the main research areas of Bacteriophage Laboratory were identification of bacterial strains from clinical specimens, selection of therapeutic phage and preparation of phage lysates, and their application in collaboration with clinics and hospitals (Duś and Ługowski, 1996). In 2000, a comprehensive review with an update on Hirszfeld Institute’s experience in phage treatment was published (Weber-Dąbrowska et al., 2000). This article provides a synopsis of clinical outcome in 1307 patients aged from 4 weeks to 86 years old treated in the years 1987–1999.

In 1999, Prof. Andrzej Górski became the Institute’s Director and later the Head of Bacteriophage Laboratory, a role he still serves. In the early 2000s, our team has formulated a new hypothesis on possible interactions between phages and the immune system including a protecting immunomodulatory role of gut phages (Górski et al., 2003; Górski and Weber-Dąbrowska, 2005). In addition, we postulated that phage can translocate from the gut and mediate their anti-inflammatory and immunomodulating activities in other organs and tissues (Górski et al., 2006). Those assumptions have been fully confirmed by the recent data of other authors (Barr et al., 2013a, b; Lehti et al., 2017; Nguyen et al., 2017; Barr, 2019). Moreover, we have further advanced our hypothesis by pointing out that various phages may mediate different immunomodulatory functions (Górski et al., 2019b).

### Phage Therapy Unit

In 2005, 1 year after Poland’s admission to the European Union, the PTU at the Hirszfeld Institute has started its activity as the first ethically approved phage treatment facility in Europe. Establishing the PTU was possible thanks to Prof. Andrzej Górski’s efforts. This center continues the rich tradition of PT in Poland, which dates to the early 1920s (Międzybrodzki et al., 2012). PTU has paved the way for compassionate use of phage therapy in modern medicine and shaped the current state of knowledge concerning the experimental use of bacterial viruses. The Phage Therapy Unit is supported by the Bacteriophage Laboratory of the Hirszfeld Institute, which carries out phage typing procedures, prepares the phage formulations for patients, and performs some other tests within experimental phage therapy. Currently, the Bacteriophage Laboratory possesses one of the largest therapeutic phage collections, which consists of over 850 described and cataloged phages against the most common bacterial human pathogens. To date, over 700 patients have been subjected to phage treatment at PTU. The phage therapy is conducted on an outpatient basis under the protocol of an experimental program, “Experimental phage therapy of drug-resistant bacterial infections, including MRSA infections,” approved by an Independent Bioethics Committee (opinion No. KB-349/2005). The program is supervised by Prof. Andrzej Górski, the head of the Phage Therapy Unit and the Bacteriophage Laboratory.

Phage scientists from the Hirszfeld Institute continue their publishing activity. In 2014, we published a book on phage therapy, *Phage Therapy: Current Research and Applications* [eds. Borysowski et al. (2014)]. The book provides comprehensive coverage of the topic with a focus on current research and emerging applications of phages and provides evidence of the unique position of the Hirszfeld Institute in the scientific world. Eric Keen from Washington University of St. Louis and Sankar Adhya from the United States Department of Health and Human Services and National Institutes of Health in their review described this position as a valuable resource for anyone interested in phage biology and/or biomedical significance that presents a compelling case that phage-based medicine is an idea whose time has come (Keen and Adhya, 2015). Recently, the second book, entitled *Phage Therapy: A Practical Approach*, has also been published by Springer [eds. Górski et al. (2019a)]. This book gives a detailed insight into the current state of the art of the therapeutic application of bacteriophages in different conditions and is, therefore, a valuable resource for individuals engaged in the medical application of novel phage therapies.

To salute the 100th anniversary of the discovery of bacteriophages and the 10th anniversary of the establishment of the Phage Therapy Unit in Wrocław, the Hirszfeld Institute organized an international conference “Clinical Phage Therapy,” held on September 26, 2015. In July 2018, the Hirszfeld Institute co-organized in Wrocław, Poland, the 5th edition of one of the biggest and most prestigious phage conferences (sponsored by the International Society for Viruses of Microbes) – Viruses of Microbes 2018 (conference leaflet available at: http://meetings.embo.org/event/18-virus-microbe). The main organizers were Prof. Krystyna Dąbrowska from our Bacteriophage Laboratory together with Prof. Zuzanna Drulis-Kawa from the University of Wrocław. This major meeting summarized the world’s current research and trends in phage applications and brought together the most prominent scientists focusing on phage research.

**FIGURE 4:**
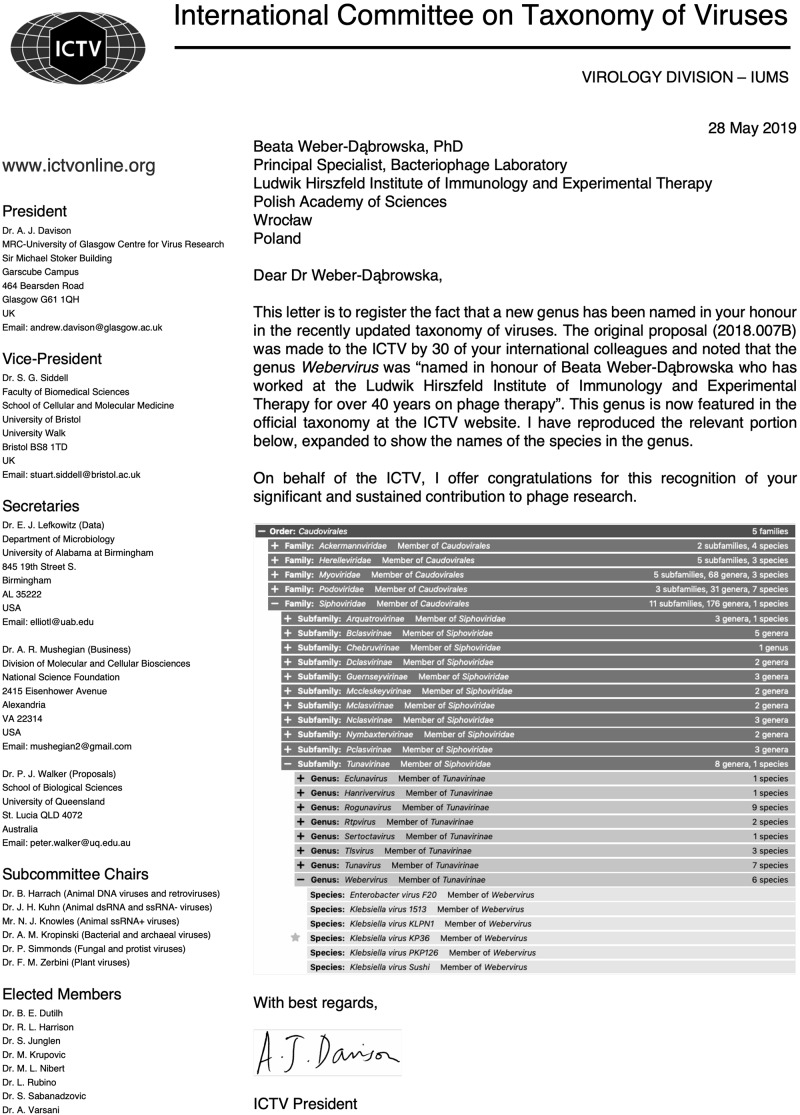
A congratulatory letter of the President of the International Committee on Taxonomy of Viruses, Dr. Andrew J. Davison to Dr. Beata Weber-Dąbrowska dated May 28, 2019; source: private archive of Dr. B. Weber-Dąbrowska.

In 2019, a longtime phage researcher, who has been working at the Hirszfeld Institute for nearly 50 years and devoted her entire career to phage, Dr. Beata Weber-Dąbrowska, has been honored in a very special way by the International Committee on Taxonomy of Viruses (ICTV). A new genus of bacterial viruses, *Webervirus*, has been named after her and is now featured in the official virus taxonomy at the ICTV website (Figure 4). Dr. Beata Weber-Dąbrowska has co-authored more than 100 articles in the phage field so far.

## Final Thoughts

Undoubtedly, establishing the PTU at the Hirszfeld Institute was a milestone in an over 100-year phage treatment tradition in Poland. These days, the PTU is the key, internationally recognized, site dedicated to experimental phage treatment, which so far has admitted hundreds of patients from all over the world.

To date, phage scientists from the Hirszfeld Institute have published over 100 articles in peer-reviewed journals (including pioneer, widely cited publications on different aspects of phage therapy in humans), submitted numerous international patent applications covering phage isolation, preparation, and application, participated in several conferences dedicated to phage research and become editors of recognized scientific books in the phage field. Despite the long tradition and achievements, we have always been very careful in assessing the effectiveness of phage treatment recognizing the fact that only clinical trials performed in line with EBM can determine this effectiveness.

In light of those facts, it is clear that Polish phage therapy has a rich and productive history. Our Phage Therapy Center has continued in its best tradition, and, based on past achievements, it has advanced the therapy by expanding the relevant knowledge with the aid of planning decisive clinical trials. At the same time, we envisage novel non-bacterial potential application of phage therapy (currently referred to as “drug repurposing”). Undoubtedly, future perspectives for phage application look no less interesting than past achievements.

The undisputed progress and significance of phage therapy in Poland would not have been possible without the contributions of Polish scientists from other centers. They did not carry out clinical phage therapy themselves, yet their basic studies and experimental work in animals have contributed greatly to the current high position of Polish phage research and therapy in the international arena. One should mention Krystyna Dąbrowska from our laboratory, Zuzanna Drulis-Kawa (University of Wrocław), Małgorzata Łobocka (Institute of Biochemistry and Biophysics, Polish Academy of Sciences in Warsaw), Jarosław Dastych (Institute of Medical Biology in Łódź), Grzegorz Węgrzyn (University of Gdańsk), and Romuald Gryko (Military Institute of Hygiene and Epidemiology in Puławy), among others. This list is certainly not exhaustive.

The survival and further development of phage research on Polish soil is the best example that science stands above political turbulences and economic obstacles, neither of which have been lacking over the past century in Poland.

## Author Contributions

MŻ wrote and revised the manuscript, reviewed available publications and documents. BW-D wrote part of the manuscript, provided unique documents regarding history of the Hirszfeld Institute along with comments and personal insight. RM and MŁ-S revised the manuscript. AG is an originator of the subject and wrote parts of the manuscript.

## Conflict of Interest

The authors declare that the research was conducted in the absence of any commercial or financial relationships that could be construed as a potential conflict of interest.
